# Dependence of rapid spike-based neural learning upon neural parameters

**DOI:** 10.1186/1471-2202-12-S1-P312

**Published:** 2011-07-18

**Authors:** David H Staelin, Keith T Herring, Carl H Staelin

**Affiliations:** 1 Research Laboratory of Electronics, Massachusetts Institute of Technology, Cambridge Massachusetts 02139, USA; 2 Hewlett-Packard Laboratories Israel, Technion City, Haifa 3200, Israel

## 

Although much is known about the physics and chemistry of neuron activity, there is still no accepted model that explains how key neural learning and computational metrics are quantitatively linked to salient neuron properties that include firing thresholds and their dynamics, dendrite structure, average firing rates, requirements for spike synchrony, dynamic range of synapse strength, synapse-atrophy dynamics, and the optimum number of synapses per neuron (~10,000). Performance metrics of interest include upper bounds on the average: 1) bits stored per neuron and per synapse, 2) extractable bits/neuron/sec and bits/spike (or bits/joule), 3) false-alarm probabilities, and 4) the time required for learning new patterns. The problem also involves how information is coded by pulses, what useful computational function is performed by a single neuron, and how that neuron learns. We propose here an Attention-Modulated Plasticity (AMP) model for neural learning that links all these neural properties to quantitative metrics. AMP performance is first predicted theoretically and then tested using time-domain simulations for two different learning models and large ensembles of plausible neural parameters. For plausible neural parameters we find: bits/neuron ≅ 200, bits/synapse ≅ 0.1, bits/spike ≅ 2, false alarm probabilities ≅ 0.2 percent, and sub-second learning.

AMP model performance metrics are derived by maximizing for given neural parameters the mutual information *L* (bits) between the ensemble  of patterns presented to the neuron during plasticity and, after training ends, the ensemble  of patterns to which that mature neuron responds with a spike. The binary vector has *Z* entries *X_i_* ∈ {0,1}, where *Z* is the number of patterns likely to be presented;  is similar and indicates both recognition successes and false alarms. The pattern associated with a spike wave incident upon a single neuron N is defined by the combination of afferent synapses it excites within a nominal 20-msec interval, together with any relative delays between those spikes observed at the somas of the input neurons; those spikes are brought into synchrony at N’s dendrites by subsequent differential delays (if any) in the intervening axons and synapse/dendrite interfaces.

AMP neurons learn a fraction *p_L_* (learning probability) of input patterns presented during plasticity, and at maturity respond to all those learned plus a fraction *p_F_* (false alarm probability) of those not presented. Plasticity is transient and triggered by attention, very likely signaled by astrocytes that respond to blood composition and to spikes from other neurons. After learning an optimum number of patterns, training ends and neurons mature in one of two modeled ways prior to testing. In the AMP synapse-atrophy (SA) model all synapses have unity strength during training and at maturity each synapse atrophies (strength → 0) if it had never been “immortalized” by an intra-neuron back-propagating pulse resulting from a coincident output spike to which that synapse contributed. In the AMP synapse-strength (SS) model the strength is binary ∈ (1,*G*), where *G* is the equivalent of being immortalized and optimum G values are found to be 1.2-1.6, depending on the assumed neural parameters; during testing (maturity) the firing threshold *H* temporarily increases to *GH*. Other key assumed neural parameters include the fraction *R*^-1^ of patterns for which a given neuron spikes, the number *C* of dendrite compartments capable of independently firing and back-propagating reinforcement spikes, and the number *D* of assumed distinguishable delays within a single spike wave. Both *C* and *D* can be unity.

The many conclusions include: 1) if a single spike implies only that its stimulating pattern had been learned earlier, then:  bits/neuron,

2) *L* = 0 for this metric unless *p_F_* <*p_L_*; for reasonable assumptions this requires that there be distinct plastic and mature neural states, where maturity might involve either atrophy (SA model) or *H* → *GH* (SS model), 3) *L*(SA) ≅ 0.2 *D*^1.4^*R*^0.9^*C*^0.7^*H*^0.7^ bits/neuron, 4) bits per strong synapse ≅ 0.1 ≅ 2/*H*^0.8^, 5) SA outperforms SS by factors of ~2-8 while SS more readily interleaves plasticity with recognition, 6) for plausible neural parameters L is maximized by ~10,000 synapses, 7) optimum *R* values apparently trade bits/neuron against bits/neuron/sec, 8) optimum *H* values apparently trade the metabolic costs of neurons vs. synapses, 9) both AMP SA and SS learning can sequentially train multiple neural layers; as little as one training exposure may suffice per pattern, and 10) both learning models can train neural layers with rich top-down feedback synapses that arguably permit increased noise immunity, source data compression, recognition of sequential patterns, and associative memory capabilities.

